# Trabecular architecture of the medullary cavity: a comprehensive review of radiological patterns, pathological alterations

**DOI:** 10.1093/bjr/tqag176

**Published:** 2026-07-31

**Authors:** Shashank Chapala, Vivek Kiyawat, Jonathan Gibson, Praveen Chinniah, Shubham Arora, Ali Shah, Sumathi Vaiyapuri, Neha Nischal, Rajesh Botchu

**Affiliations:** Department of Radiology, AIG Hospitals, Hyderabad, 500032, India; Department of Musculoskeletal Radiology, University Hospitals of North Midlands, Stoke on Trent, ST4 6QG, United Kingdom; College of Medicine and Dental Sciences, University of Birmingham, Birmingham, B15 2TT, United Kingdom; Emergency and Trauma Radiology, Sunnybrook Health Sciences Centre, University of Toronto, Toronto, ON M4N 3M5, Canada; Department of Radiology, Max Super Speciality Hospital, New Delhi, 110017, India; Department of Musculoskeletal Radiology, Nottingham University Hospital NHS Trust, Nottingham, NG5 1PB, United Kingdom; Department of Musculoskeletal Pathology, University Hospitals Birmingham NHS Trust, Birmingham, B15 2GW, United Kingdom; Department of Radiology, Holy Family Hospital, New Delhi, 110025, India; Department of Musculoskeletal Radiology, Royal Orthopaedic Hospital, Birmingham, B31 2AP, United Kingdom

**Keywords:** trabecular bone, medullary cavity, bone density, Singh index, photon-counting CT, opportunistic CT

## Abstract

The trabecular bone of the medullary cavity is a complex 3D lattice essential for skeletal load distribution and metabolic homeostasis. This article provides a narrative review of trabecular architecture, ranging from hierarchical nanoscopic fibrils to macroscopic stress trajectories. We analyze the radiological appearance of normal and pathological alterations, including thickening, breakage, faintness, rarefaction, and infiltration. Modalities such as high-resolution MRI, dual-energy CT, and the emerging photon-counting detector CT are evaluated for their diagnostic precision. Furthermore, we examine clinical indices like the Singh Index and the growing role of opportunistic CT screening. Understanding these morphological transitions is critical for the early detection of systemic metabolic diseases and the monitoring of localized mechanical failure.

## Introduction

Trabecular bone, also known as cancellous or spongy bone, constitutes approximately 20% of the human skeleton but possesses a remodeling rate significantly higher than that of cortical bone.^1^ Occupying the medullary cavity and the meta-epiphyseal regions of long bones, this intricate 3D lattice serves as a vital structural and metabolic organ. It acts as a biomechanical buffer, transferring mechanical loads from articular surfaces to the cortical shell, while providing a sophisticated environment for load distribution, shock absorption, and hematopoiesis.^2^

Historically, bone health was assessed primarily via bone mineral density (BMD), but recent research underscores that microarchitecture is an independent and vital determinant of bone strength.^3^^,^ ^4^ Deterioration of this architecture—characterized by a shift from robust plate-like structures to fragile rod-like ones—is a hallmark of aging and metabolic bone diseases such as osteoporosis.^5^

While existing literature frequently addresses isolated bone pathologies, this review uniquely synthesizes multimodality imaging approaches to provide a consolidated, symptom-based framework for interpreting morphological transitions in the medullary cavity. By correlating advanced imaging with underlying biomechanics, we aim to enhance the radiologist’s ability to differentiate between physiological stress adaptations and pathological deterioration.

## Search strategy and methodology

This narrative review was conducted by searching PubMed, Scopus, and Google Scholar for literature published between 2000 and 2026. The search utilized MeSH terms and keywords including “Trabecular Bone,” “Bone Microarchitecture,” “Photon-Counting CT,” “Dual-Energy CT,” “Bone Marrow Edema,” and “Opportunistic CT.” Articles were selected based on their clinical relevance, focus on radiological imaging of the medullary cavity, and peer-reviewed status. Older seminal papers defining fundamental biomechanical laws were also included for historical context.

## Hierarchical structural organization: from nanoscale to macroscopic texture

The complexity of bone tissue is best understood through its hierarchical organization, which encompasses as many as 12 distinct structural levels^6^ (Figure 1: Schematic of hierarchical bone architecture).

**Figure 1:**
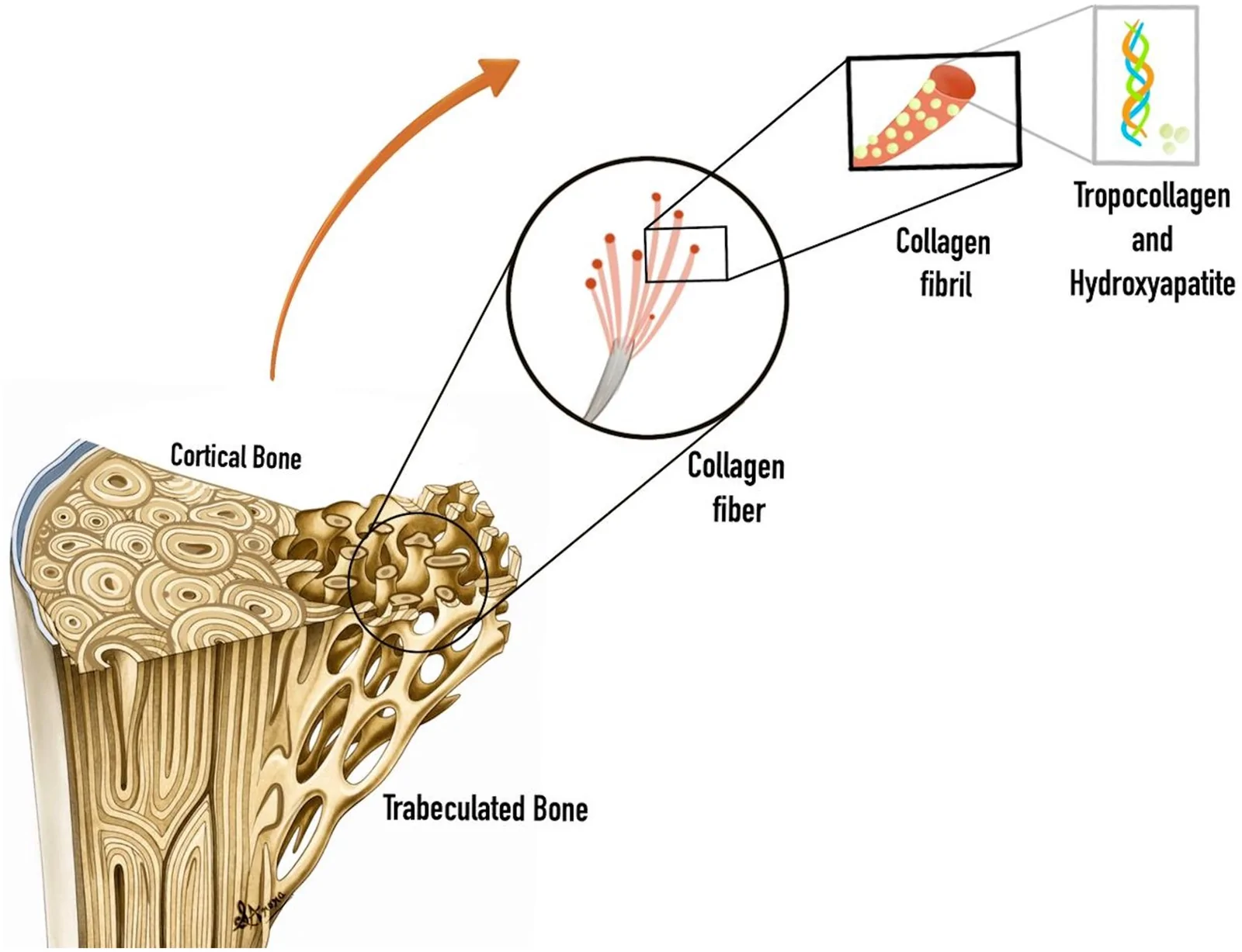
Schematic of normal bone trabecular architecture depicting the hierarchical organization from nanoscale collagen fibrils to macroscopic stress trajectories.

At the nanoscopic level, the fundamental building blocks are mineralized collagen fibrils, measuring between 1 and 100 nm.^2^^,^ ^6^ These fibrils consist of Type I collagen molecules interspersed with hydroxyapatite crystals. The spatial arrangement of these fibrils dictates the subsequent levels of organization, including the formation of mineral ellipsoids—micrometer-sized structures reported in healthy and diseased cortical and trabecular bone that contribute to the overall mineralization pattern.^7^ Progressing to the microscopic level, these fibrils organize into lamellae. Within this lamellar matrix, osteocyte lacunae house the sensing cells responsible for bone remodeling, connected via a dense lacuno-canalicular network (LCN).^8^^,^ ^9^

The mesoscopic level is defined by the individual trabecular units, which typically manifest as plates or rods. In a young, healthy individual, the network is predominantly plate-like and plexiform. The transition from a robust, plate-dominated network to a fragile, rod-dominated one is a hallmark of aging and metabolic bone disease.^3^^,^ ^5^ The macroscopic texture of the medullary cavity is the result of these units organizing into a highly anisotropic network. This anisotropy is strictly aligned with the principal stress trajectories experienced by the bone during physiological loading, a principle formalized as Wolff’s Law^10^ (Table 1).

**Table 1:** Hierarchical levels and clinical features of trabecular bone.

Hierarchical level	Approximate scale	Primary components	Clinical and functional significance
**Nanoscale**	1-100 nm	Collagen fibrils, mineral crystals	Fundamental tensile strength
**Microscale**	1-100 μm	Lamellae, osteocytes, canaliculi	Biological signaling, bone remodeling
**Mesoscale**	100 μm-1 mm	Trabecular plates and rods, osteons	Structural stability (site of osteoporotic structural shifts)
**Macroscale**	>1 mm	Trabecular network, medullary cavity	Global load distribution and shock absorption

## Biomechanical determinants of trabecular configuration

The architecture of the medullary cavity is a living record of the mechanical stresses applied to the skeleton. The distribution of trabeculae is optimized to provide maximum biomechanical adaptation for a minimal material mass.

### Tensile and compressive systems of the proximal femur

The internal architecture of the femoral neck and head is organized into 2 major systems.^11^ The medial system (principal compressive group) consists of vertically oriented trabeculae designed to withstand the compressive forces of body weight. The lateral system (principal tensile group) forms an arc counteracting the tensile forces generated by the bending of the neck. The central region where these systems intersect defines a neutral axis where forces reach equilibrium, housing Ward’s Triangle, an area of relative rarefaction highly susceptible to osteoporotic bone loss (Figure 2).

**Figure 2:**
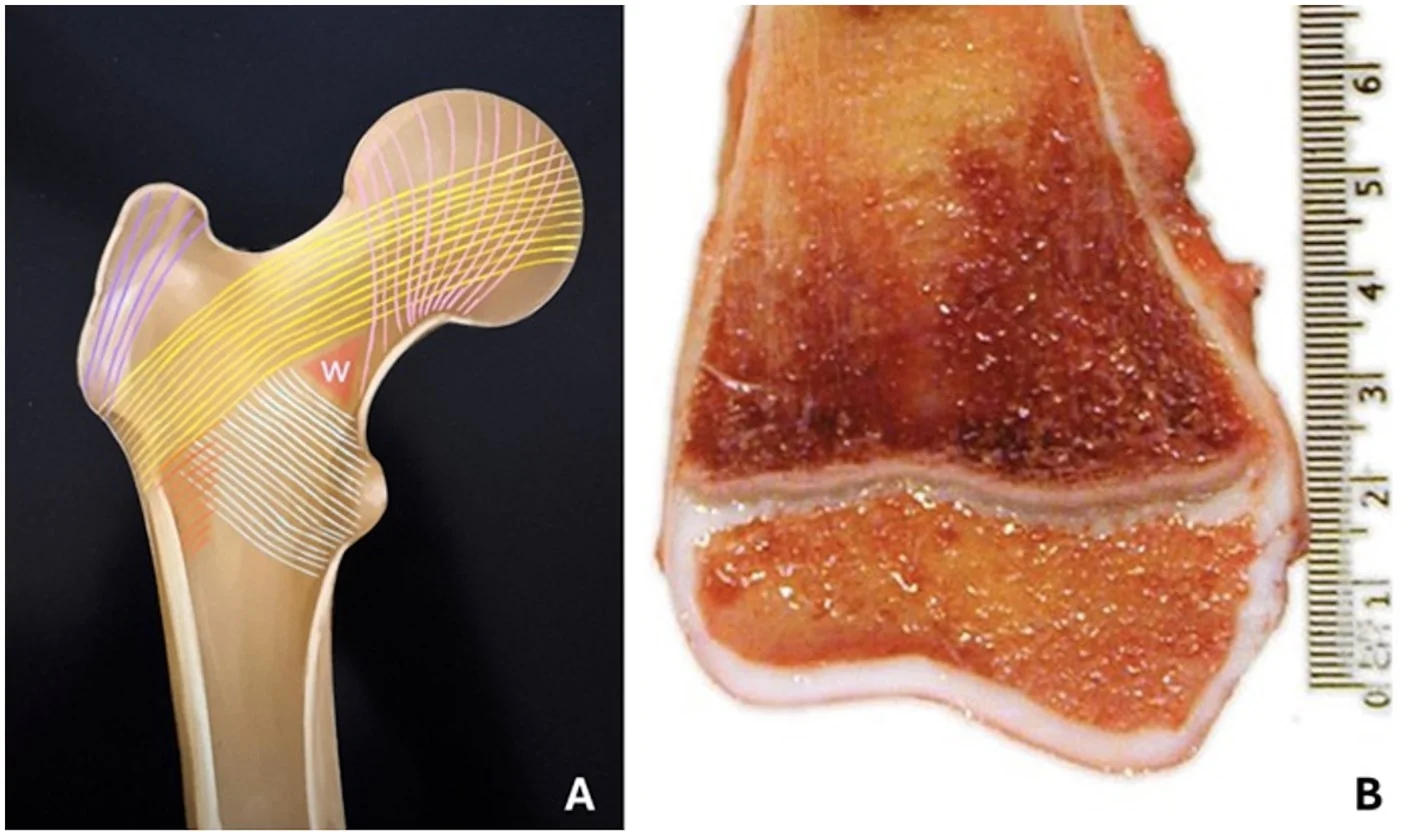
Biomechanical failure of the proximal femur. Subcapital fracture bisecting the primary tensile and compressive trabecular groups, resulting in displacement. (A) A schematic to show the biomechanical failure. (B) A real-life image of a subcapital fracture.

### Adaptation to weight-bearing and impact

Trabecular adaptation is highly sensitive to the magnitude and frequency of loading. High-impact loading, such as repetitive stress experienced by athletes, triggers a physiological thickening of the trabeculae.^12^^,^ ^13^ This adaptive thickening serves to enhance the mechanical resistance of the medullary cavity but may eventually lead to subarticular stress reactions if the loading outpaces the bone’s remodeling capacity.^14^

## Radiological modalities for medullary assessment

The noninvasive assessment of trabecular microstructure requires imaging tools that can resolve features on the scale of 50-100 μm.

### Conventional radiography and texture analysis

Plain radiographs are the most widely available tool for screening. However, they require a 30%-50% loss of bone mineral density before lytic lesions become apparent. Trabecular Bone Texture Analysis (TBTA) utilizes digital algorithms to quantify the roughness and directionality of the trabecular pattern,^15^ differentiating healthy bone from early stages of osteoarthritis.^16^^,^ ^17^

### CT: PCD-CT and DECT

Photon-Counting Detector CT (PCD-CT) converts X-ray photons directly into electrical signals, allowing for significantly higher spatial resolution and reduced electronic noise compared to conventional energy-integrating detectors (Figure 3). While evaluating true “individual trabeculae” at a microscopic level remains challenging in routine clinical practice, PCD-CT currently offers unprecedented sub-millimeter visualization of trabecular networks and microfractures at a reduced radiation dose.^18–22^ Dual-energy CT (DECT) has revolutionized the detection of bone marrow edema (BME).^23–25^ By utilizing material decomposition to create “virtual non-calcium” images, DECT suppresses the high attenuation of mineralized bone, visualizing the underlying BME. This is highly valuable in emergency settings for occult fractures (Figure 4 and Table 2).

**Figure 3:**
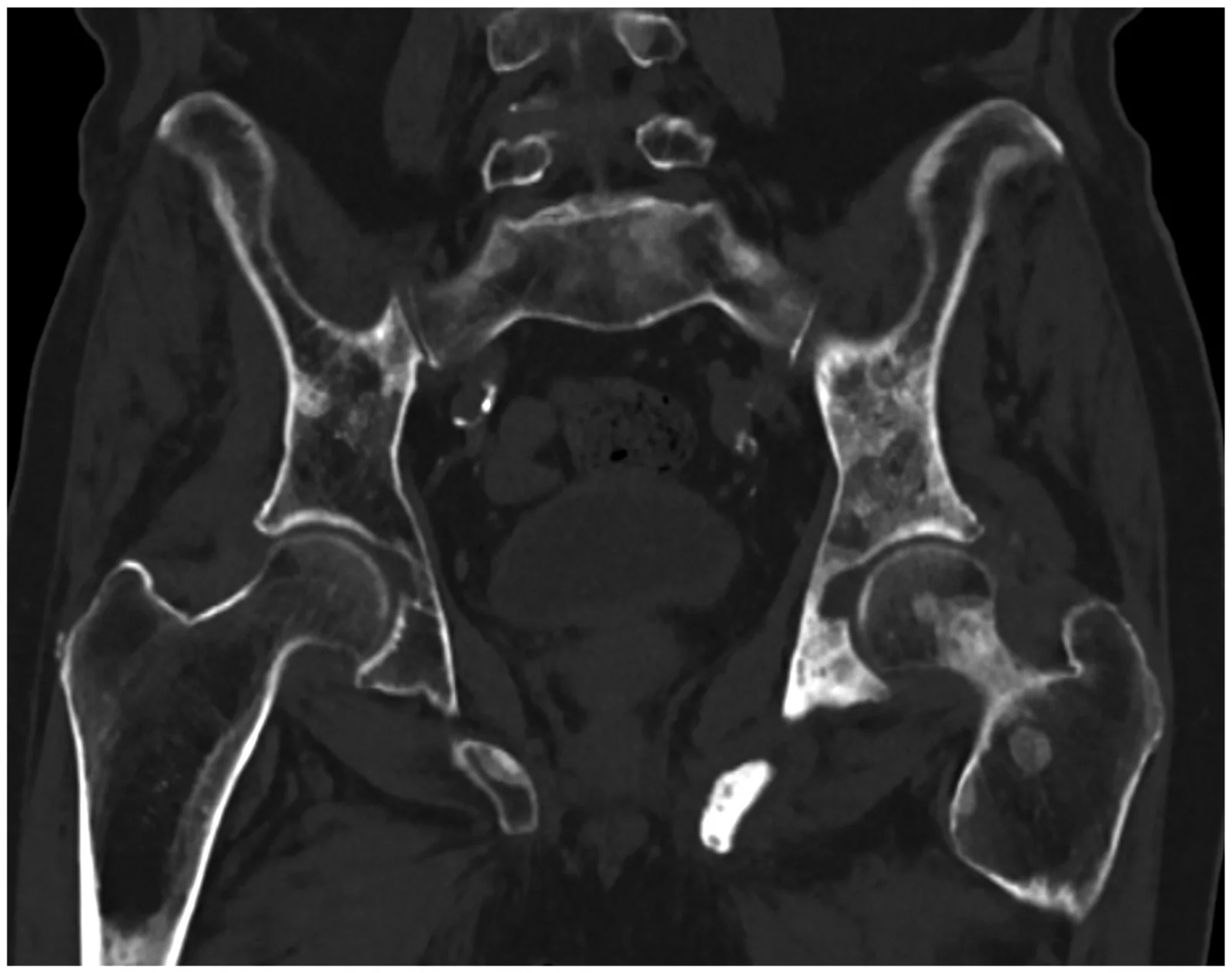
Coronal PCD-CT reconstruction of the pelvis showing sclerotic metastatic deposits. The high spatial resolution highlights the infiltration pattern and disorganized osteoblastic activity.

**Figure 4:**
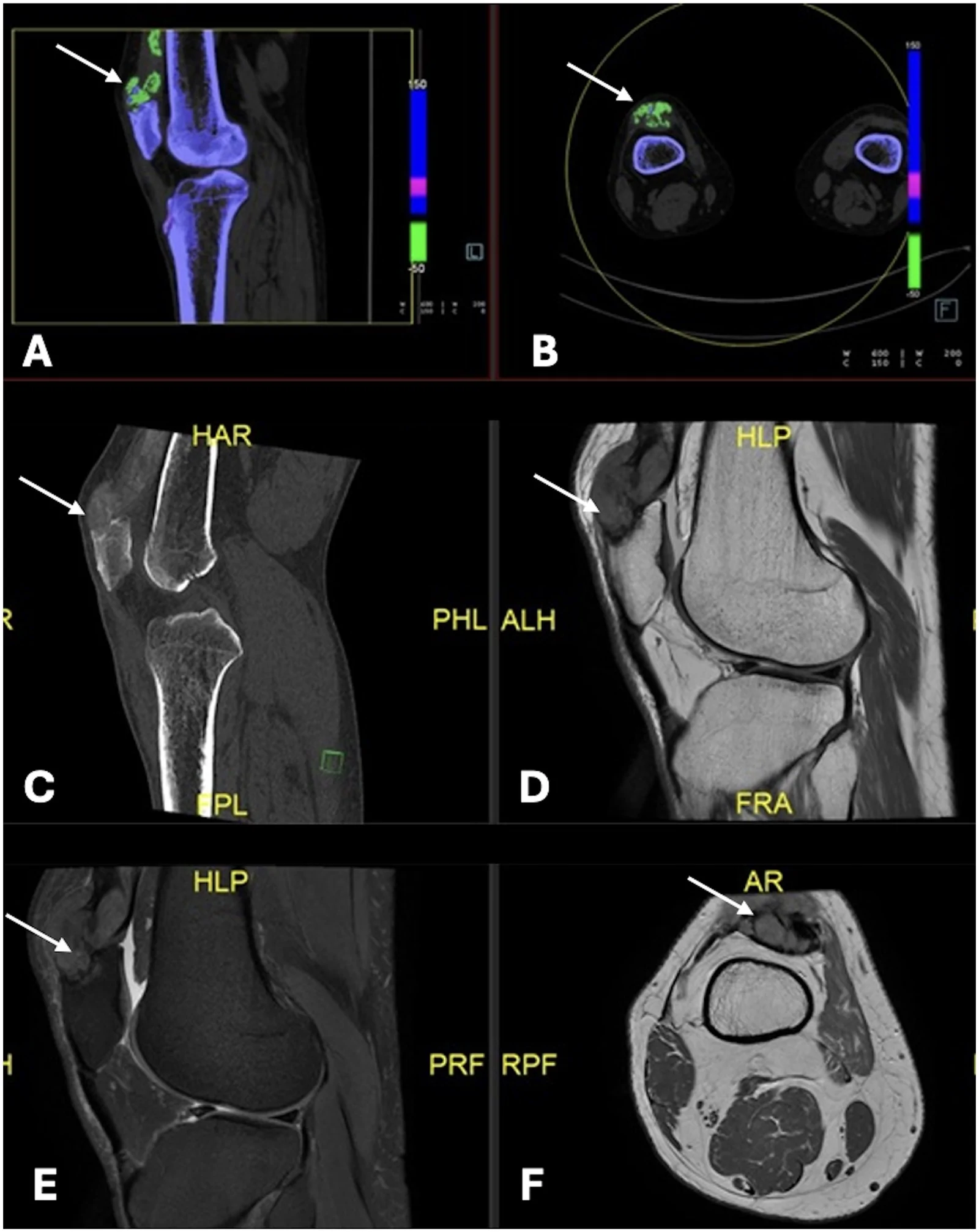
Multimodal imaging in chronic tophaceous gout. (A) DECT material decomposition highlights urate deposition. (B) Sagittal CT shows a well-defined, “punched-out” erosion. (C-F) MRI demonstrates marrow replacement by the tophus.

**Table 2:** CT modality comparison and clinical utility.

CT modality	Detector pixel size	Key technical advantage	Primary clinical application
Conventional (EID-CT)	0.5-0.6 mm	Fast, widely accessible	Evaluating gross fractures and preoperative planning
Photon-counting (PCD-CT)	0.2-0.25 mm	Ultra-high resolution	Visualizing microfractures and fine trabecular detail
Dual-energy (DECT)	Varies	Material decomposition (VNC)	Detecting occult bone marrow edema (BME) and urate mapping

### MRI: UTE and HR-MRI

MRI is the gold standard for assessing marrow infiltration. High-resolution MRI (HR-MRI) indirectly visualizes trabeculae as dark structures surrounded by bright marrow signal.^4^ Ultrashort echo time (UTE) MRI sequences employ TEs in the range of 10-100 μs, enabling the direct detection of signal from bound water within the bone matrix.^26^^,^ ^27^ While highly promising for estimating bone volume fraction noninvasively, UTE-MRI is currently utilized predominantly in research settings rather than routine clinical practice.

## Pathological alterations of trabecular architecture

The trabecular network responds to insults through a limited set of morphological changes. By categorizing these changes radiologically, clinicians can systematically narrow the differential diagnosis (Table 3).

**Table 3:** Trabecular alteration patterns and differential diagnosis.

Alteration pattern	Radiological description	Key clinical differentials
Thickening	Coarsened trabeculae, ivory bone	Paget’s disease, osteoblastic metastases, chronic osteomyelitis
Thinning	Fine, attenuated trabeculae	Osteoporosis, steroid use, disuse atrophy
Breakage	Linear fracture lines, cortical step-off	Subchondral insufficiency fractures, trauma
Faintness	Obscured trabecular detail by edema (BME)	Stress reactions, early osteonecrosis
Infiltration	Marrow signal change with preserved bone architecture	Early myeloma, leukemia, lymphoma
Destruction	Lytic gaps, “punched-out” lesions	Aggressive malignancies, acute osteomyelitis

### Thickening and sclerosis: the hypertrophic response

Thickening of the trabeculae (“sclerosis”) occurs when bone formation outpaces resorption.

- Paget’s disease: Characterized by a focal disturbance in bone remodeling resulting in expanded, coarsened bone with thickened, disorganized trabeculae^28^ (Figure 5).
- Osteoblastic metastases: Neoplasms such as prostate cancer trigger an osteoblastic response, leading to focal or diffuse trabecular thickening, appearing as low-signal intensity on T1/T2 MRI.^29^

**Figure 5:**
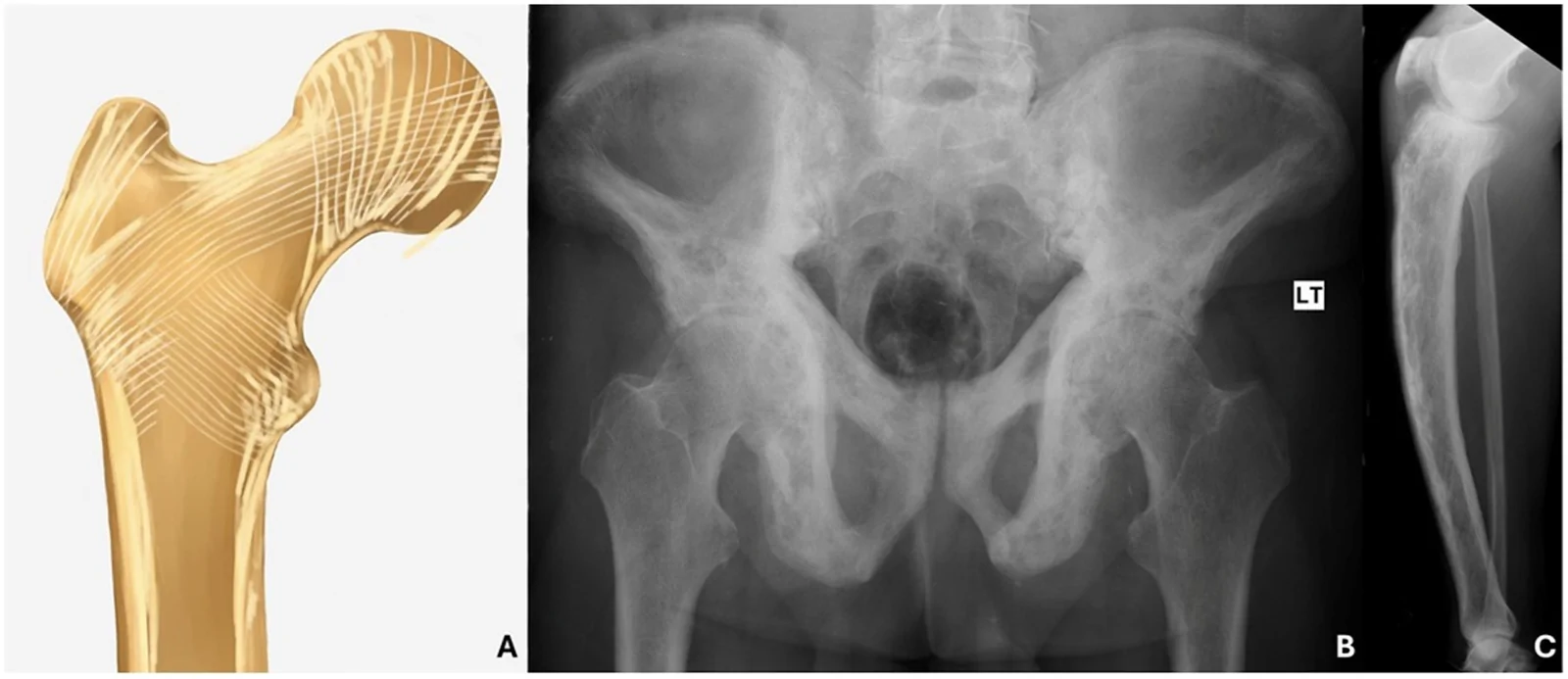
Paget’s disease. Schematic (A), AP pelvic (B), and lateral (C), tibial radiographs demonstrate classic cortical thickening, osseous expansion, and coarsened trabecular patterns.

### Thinning and rarefaction: the atrophic response

Rarefaction involves a decrease in the number and thickness of trabecular units.

- Osteopenia/osteoporosis: Systemic generalized reduction in bone mass. Loss of estrogen leads to the perforation of trabecular plates and conversion into thin rods^3^^,^ ^4^ (Figure 6).
- Disuse atrophy: Immobilization leads to immediate bone loss mediated by osteocytes increasing the secretion of sclerostin (Figure 7).

**Figure 6:**
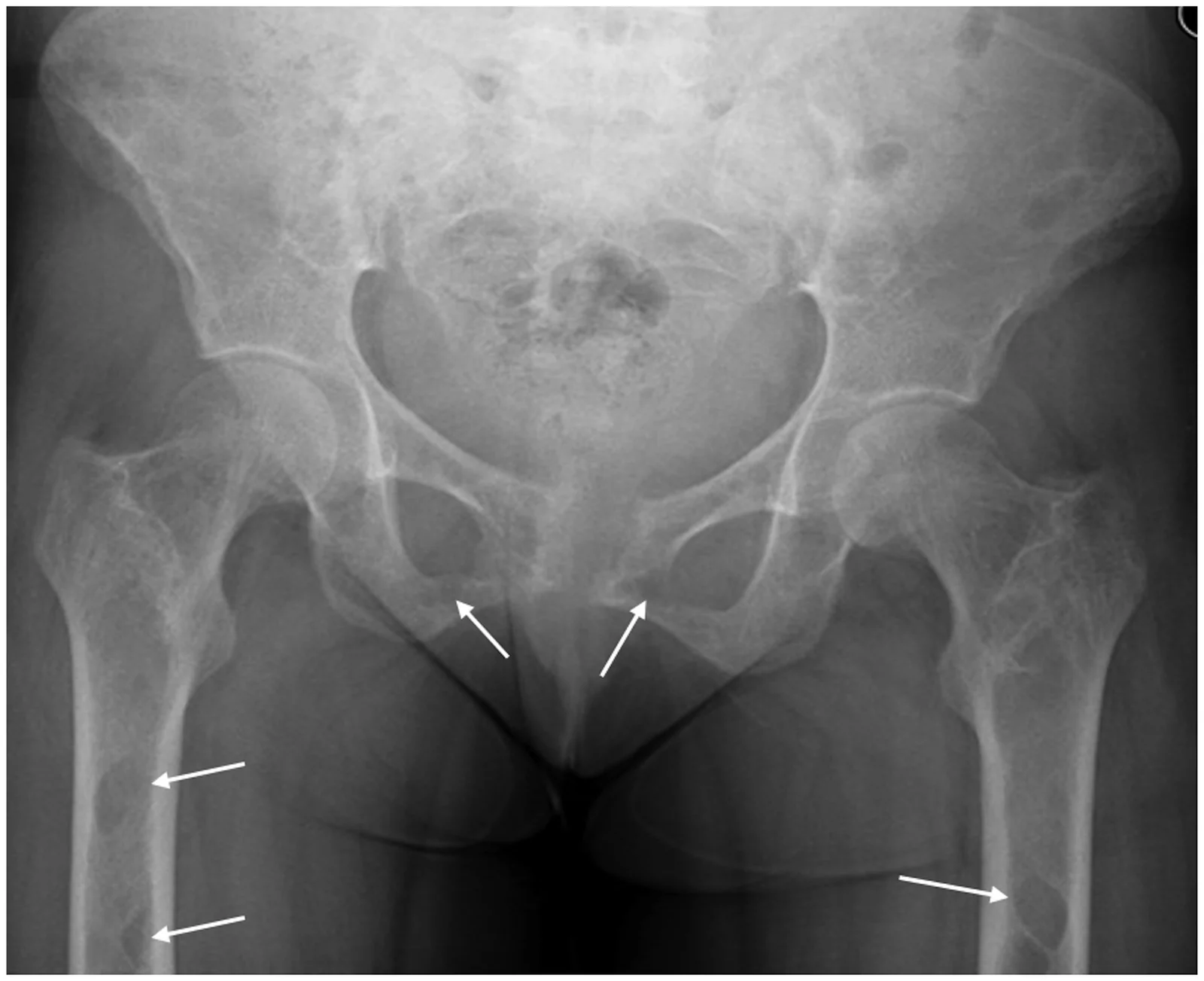
AP radiograph demonstrating lytic lesions (brown tumors) in hyperparathyroidism, indicating focal osseous resorption and structural compromise.

**Figure 7:**
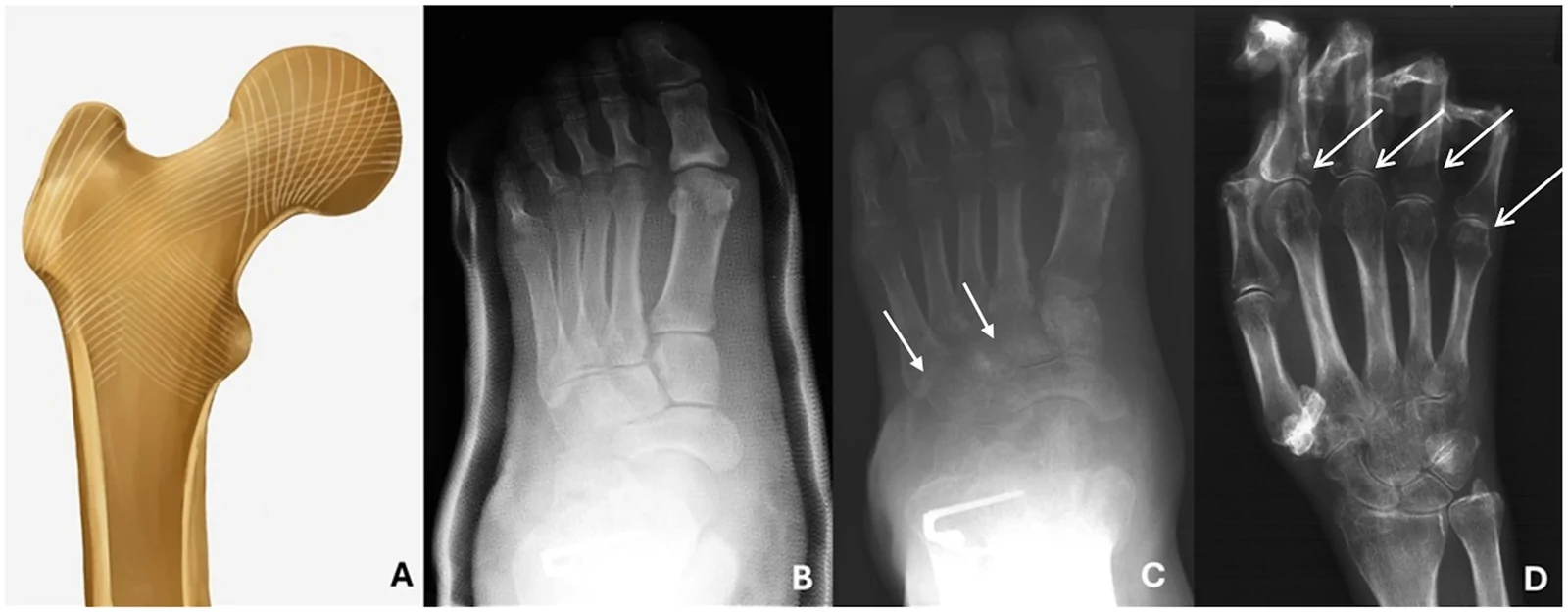
Rapid trabecular attenuation. (A) Schematic of rapid trabecular attenuation. Radiographs show regionalized osteopenia and thinning following cast immobilization (B and C) and in systemic sclerosis (D).

### Breakage and faintness: the stress-response spectrum

- Subarticular stress response: Reactive changes in subchondral bone secondary to repetitive stress without a discrete fracture line, manifesting as ill-defined BME on fluid-sensitive MRI (Figures 8 and 9).
- Subchondral insufficiency fracture (SIF): A stress fracture in abnormally weakened bone, showing a curvilinear low-signal intensity line centered in the trabecular bone surrounded by edema^29^ (Figure 10).

**Figure 8:**
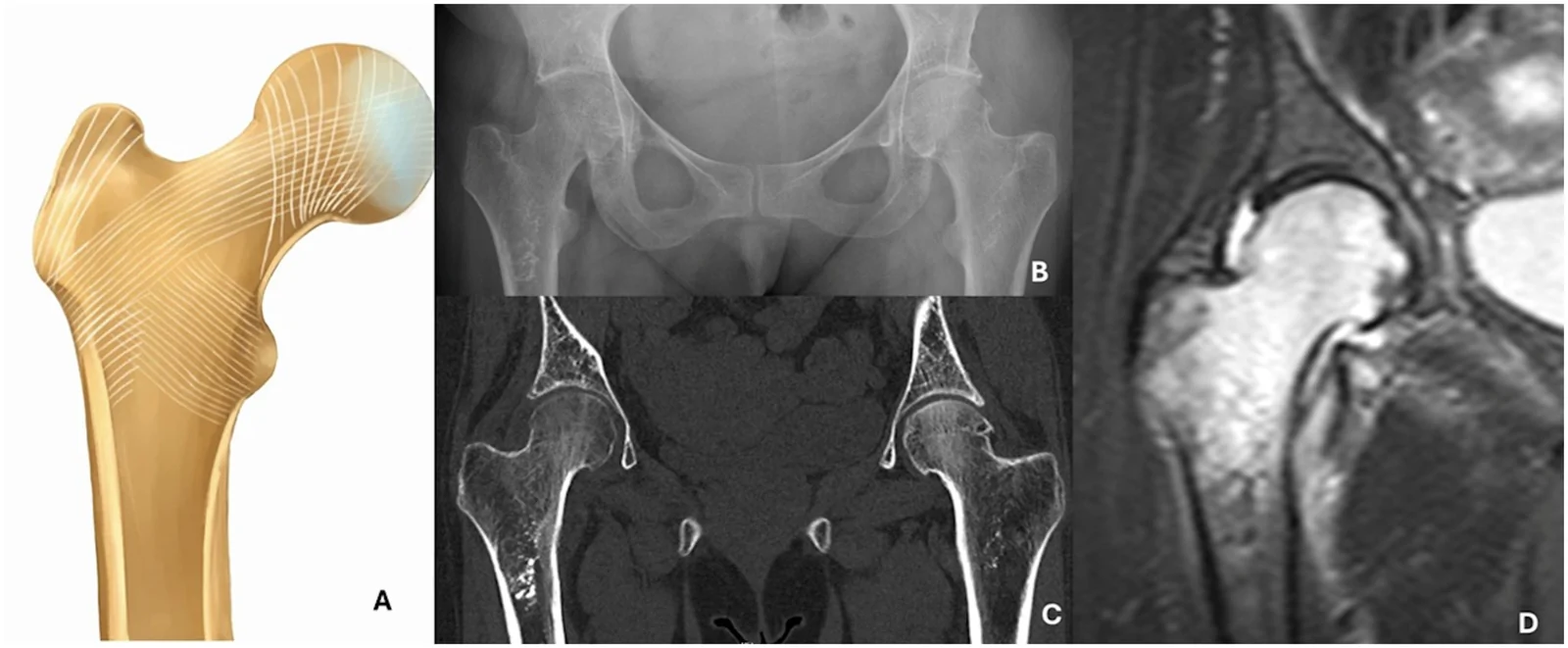
Transient osteoporosis of the hip. Schematic (A), AP X Ray (B) and MRI (C and D) demonstrates extensive, diffuse bone marrow edema within the femoral head, obscuring fine trabecular detail.

**Figure 9:**
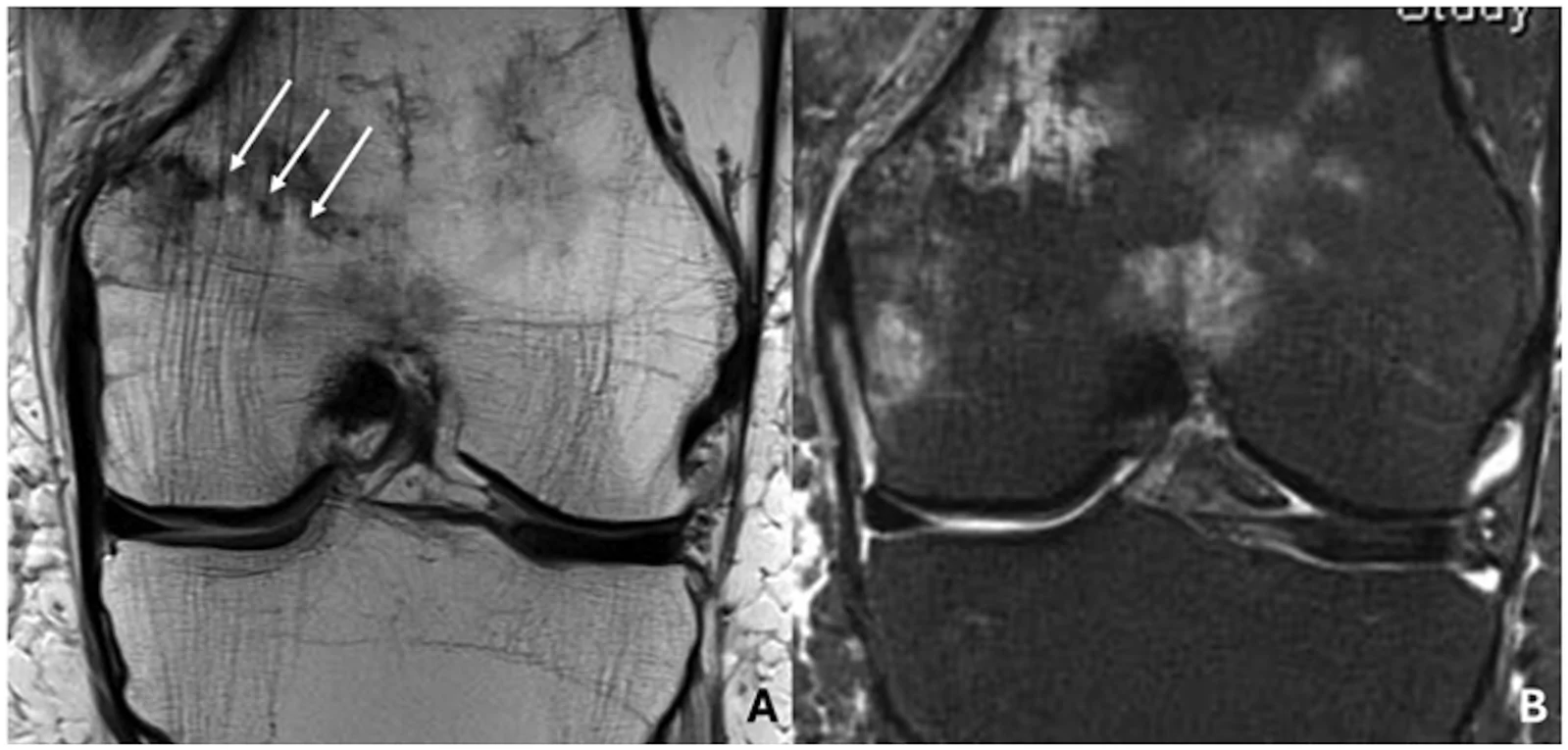
Coronal MRI of a stress reaction. Fluid-sensitive sequences show extensive hyperintense marrow edema indicative of micro-architectural disruption without a discrete fracture line. This is shown in both Figure A and B.

**Figure 10:**
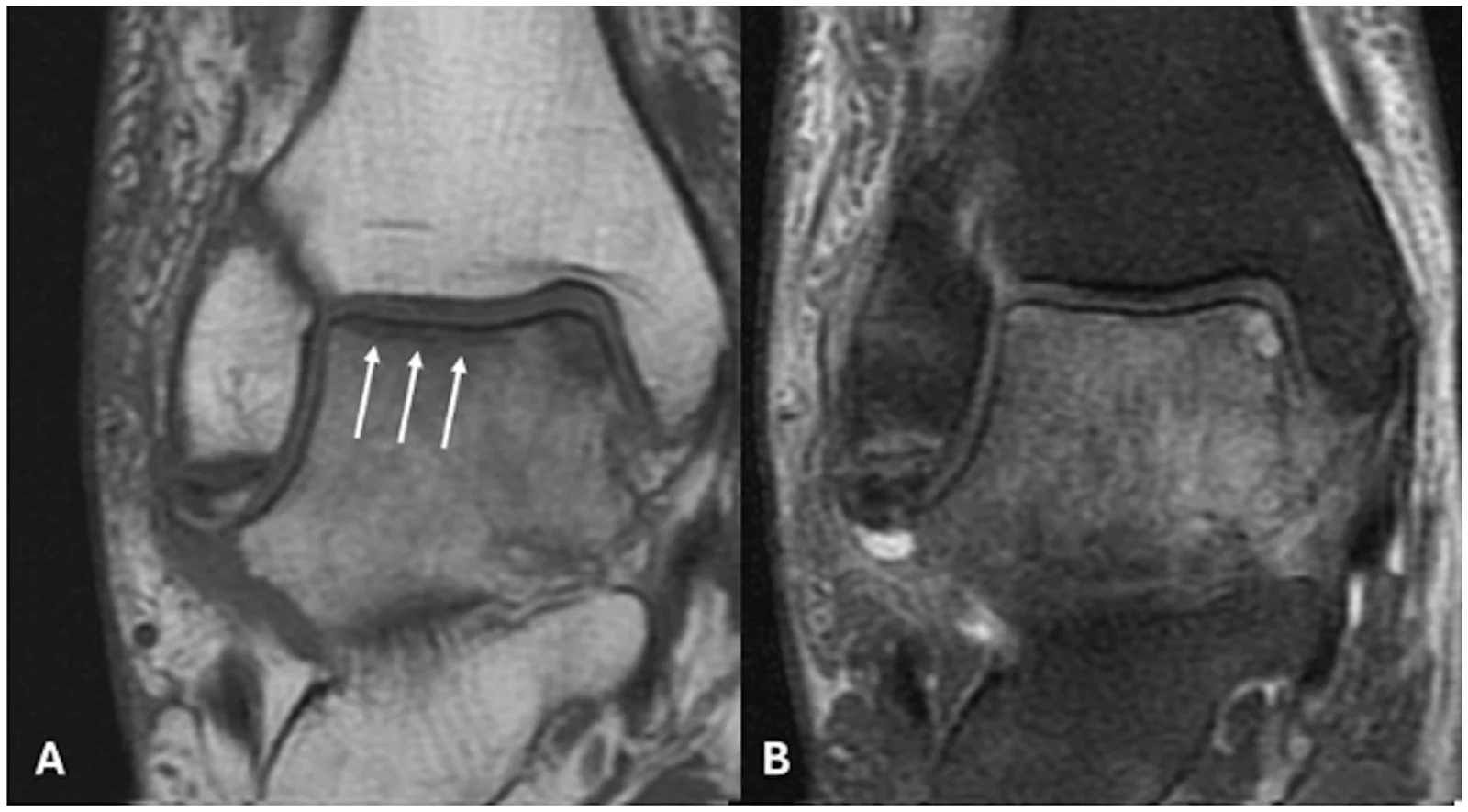
Subchondral insufficiency fracture (SIF) of the talus. Coronal T1 MRI reveals a horizontal low-signal line of trabecular collapse with surrounding reactive edema. This is shown in both Figure A and B.

### Infiltration and destruction: the aggressive pattern

- Multiple myeloma: Infiltrates the marrow space, initially without immediate trabecular destruction (best seen on MRI as focal/diffuse low T1 signal),^30^ progressing to “punched-out” lytic lesions.^25^
- Lytic metastatic disease: Causes aggressive trabecular destruction with ill-defined margins and a lack of reactive sclerosis (Figure 11).

**Figure 11:**
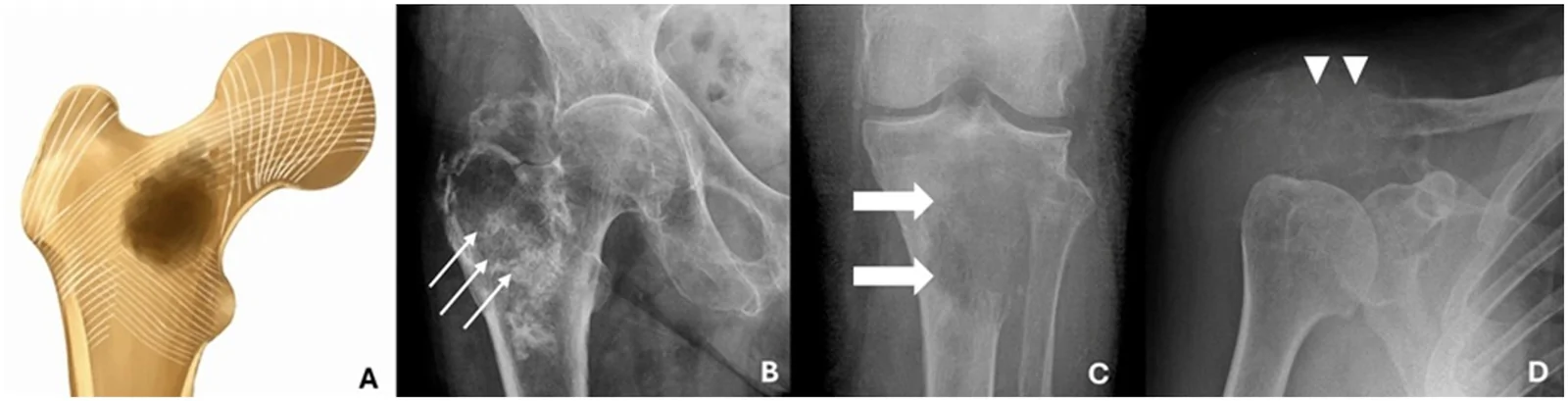
Aggressive lytic lesions schematic (A). Radiographs demonstrate permeative destruction of the organized trabecular network in chondrosarcoma (B) and renal cell carcinoma (C) and in the shoulder (D).

## Differential diagnosis: focal vs diffuse and local vs systemic

A critical step in the radiological assessment of the medullary cavity is determining the distribution of trabecular loss or alteration. This distinction is the primary for narrowing the differential diagnosis.

### Focal and localized alterations

Focal alterations are confined to a single bone or a specific joint compartment. These are typically the result of local factors: (1) trauma: osseous contusions and fractures follow specific mechanical patterns. (2) Focal infection: a Brodie’s abscess or early osteomyelitis may be localized to the metaphysis. (3) Primary neoplasms: lesions like chondroblastoma (found in the epiphyses of children) or giant cell tumors (found in the epiphyses of adults) are characteristically focal. (4) Benign lesions: vertebral hemangiomas present with a classic “polka-dot” or “corduroy” pattern due to coarsened vertical trabeculae.

### Diffuse and systemic alterations

Diffuse alterations involve the entire skeleton or multiple noncontiguous sites, suggesting an underlying systemic cause: (1) Metabolic bone disease: osteoporosis and hyperparathyroidism cause generalized rarefaction. (2) Hematologic malignancies: leukemia and diffuse multiple myeloma infiltration affect the entire medullary system. (3) Systemic osteonecrosis: often seen in patients with sickle cell disease or long-term steroid use, this can manifest as multifocal infarcts in the marrow of long bones.

### Descriptive logic for diagnostic flow

Assess multiplicity: Are the lesions solitary (focal) or distributed throughout the skeleton (? diffuse). Solitary: Consider local trauma, primary tumor, or focal infection; Diffuse: Consider metabolic disease, systemic malignancy, or generalized marrow infiltration.

Evaluate for BME: Present: Suggests an acute process (trauma, infection, stress response) or an active inflammatory state; Absent: Suggests a chronic or stable process (atrophy, healed fracture, benign dysplasia).

Determine the character of loss: Nondestructive infiltration: Normal trabecular pattern is maintained despite marrow signal change. This is highly suggestive of multiple myeloma or leukemia.; Lytic destruction: Loss of both marrow and trabecular units. This indicates an aggressive process like malignancy or acute infection.

## The Singh index and modern clinical practice

The Singh index (SI), introduced in 1970, remains a classic tool for the semi-quantitative assessment of osteoporosis using plain radiographs of the proximal femur.^31^ It evaluates the predictable sequence in which trabecular groups disappear as BMD decreases. While valued for its simplicity, its inter-observer reliability is often criticized^32^^,^ ^33^ (Table 4).

**Table 4:** Singh index grades for proximal femur assessment.

Singh index grade	Radiological appearance	Clinical status
Grade VI	All normal trabecular groups are visible	Normal bone density
Grade V	Principal tensile and compressive trabeculae are prominent	Normal bone density
Grade IV	Markedly reduced tensile trabeculae	Mild osteopenia
Grade III	Break in continuity of tensile trabeculae	Definite osteoporosis
Grade II	Only compressive trabeculae remain prominent	Severe osteoporosis
Grade I	Compressive trabeculae are markedly reduced	Severe osteoporosis

Modern clinical guidelines definitively favor dual-energy X-ray absorptiometry (DXA)-based BMD and the trabecular bone score (TBS) for diagnosing osteoporosis. Furthermore, opportunistic CT screening has emerged as a powerful modern adjunct. Utilizing routine abdominal or pelvic CT scans performed for other indications, automated software can assess trabecular attenuation (in Hounsfield Units) of the lumbar spine. This opportunistic approach has shown high diagnostic accuracy for bone mineral density assessment, proving particularly valuable in pre-surgical planning and monitoring vulnerable populations, such as patients following adult and pediatric liver transplantation.^34^^,^ ^35^ However, the SI remains a useful screening tool in resource-limited environments.

## Conclusion

The analysis of trabecular architecture within the medullary cavity reveals a dynamic hierarchical system responsive to mechanical, metabolic, and pathological stimuli. The transition from plate-like to rod-like morphology serves as a sentinel sign of skeletal aging, while focal alterations such as sclerosis and destruction provide diagnostic markers for malignancy. Advanced technologies such as PCD-CT offer unprecedented precision in quantifying bone micro-geometry. By integrating these multiscale morphological and radiological findings, clinicians can more accurately predict skeletal stability and prevent the progression of irreversible joint failure.

## Data Availability

Data is available to share on request.
